# Pet Attachment and Anxiety and Depression in Middle-Aged and Older Women

**DOI:** 10.1001/jamanetworkopen.2024.24810

**Published:** 2024-08-01

**Authors:** Magdalena Żebrowska, Susanne Strohmaier, Curtis Huttenhower, A. Heather Eliassen, Oana A. Zeleznik, Carri Westgarth, Tianyi Huang, Francine Laden, Jaime E. Hart, Bernard Rosner, Ichiro Kawachi, Jorge E. Chavarro, Olivia I. Okereke, Eva S. Schernhammer

**Affiliations:** 1 Channing Division of Network Medicine, Department of Medicine, Brigham and Women’s Hospital and Harvard Medical School, Boston, Massachusetts; 2 Department of Epidemiology, Center for Public Health, Medical University of Vienna, Vienna, Austria; 3 Department of Biostatistics, Harvard T.H. Chan School of Public Health, Boston, Massachusetts; 4 Department of Immunology and Infectious Diseases, Harvard T.H. Chan School of Public Health, Boston, Massachusetts; 5 Broad Institute of Harvard and MIT, Cambridge, Massachusetts; 6 Harvard Chan Microbiome in Public Health Center, Harvard T.H. Chan School of Public Health, Boston, Massachusetts; 7 Department of Epidemiology, Harvard T.H. Chan School of Public Health, Boston, Massachusetts; 8 Department of Nutrition, Harvard T.H. Chan School of Public Health, Boston, Massachusetts; 9 Department of Livestock and One Health, Institute of Infection, Veterinary and Ecological Sciences, University of Liverpool, Liverpool, United Kingdom; 10 Department of Environmental Health, Harvard T.H. Chan School of Public Health, Boston, Massachusetts; 11 Department of Social and Behavioral Sciences, Harvard T.H. Chan School of Public Health, Boston, Massachusetts; 12 Department of Psychiatry, Massachusetts General Hospital, Boston, Massachusetts

## Abstract

**Importance:**

Understanding how attachment to pets can alleviate depression and anxiety offers valuable insights for developing preventive and therapeutic strategies, particularly for those with insecure attachment styles from childhood trauma.

**Objective:**

To determine if a close bond with a pet is associated with reduced depression and anxiety, especially among women who experienced childhood abuse.

**Design, Setting, and Participants:**

This cross-sectional study involved women who voluntarily enrolled in the Mind Body Study (MBS), a substudy of the Nurses’ Health Study II (NHS2) focusing on psychosocial factors. Women reporting childhood abuse were oversampled to capture their psychosocial distress in adulthood. MBS participants were invited to complete comprehensive online questionnaires, which were administered twice (March 2013 and February 2014).

**Exposure:**

Pet attachment measured by Lexington Attachment to Pets Scale (LAPS).

**Main Outcomes and Measures:**

Levels of depression and anxiety (10-item Centre for Epidemiologic Studies Depression Scale [CESD-10]; Kessler Psychological Distress Scale [K6]; 7-item Generalized Anxiety Disorder scale [GAD-7]; Crown Crisp Experiential Index phobic anxiety subscale [CCI]), considered individually and combined into an overall *z*-score measure of anxiety and depression symptoms.

**Results:**

A total of 214 women (mean [SD] age, 60.8 [3.9] years) were included; 156 women (72.6%) reported a history of childhood abuse. Of 688 invited MBS participants in 2013, 293 (42.6%) expressed interest; there were 228 completed questionnaires (response rate, 77.8%) in 2013 and 208 questionnaires (response rate, 71.0%) in 2014. LAPS scores were provided by 140 participants (65.4%), 78 (55.7%) for dogs and 46 (32.9%) for cats. Overall higher pet attachment on the LAPS score was significantly associated with lower GAD-7 scores (β = −0.17; 95% CI, −0.29 to −0.06), but there was no association for phobic anxiety or depression. There were no statistically significant associations between cat attachment and depression or anxiety. Higher dog attachment was associated with significantly lower scores in depression (CESD-10: β, −0.47; 95% CI, −0.68 to −0.26; K6: β = −0.42; 95% CI, −0.54 to −0.31), generalized anxiety (GAD-7: β = −0.47; 95% CI, −0.65 to −0.3), and the overall measure of anxiety and depression (*z* score: β = −0.12; 95% CI, −0.17 to −0.08), but there was no association between dog attachment and phobic anxiety (CCI: β = −0.08; 95% CI, −0.24 to 0.09). All effect sizes for associations were higher when analyses were restricted to women with a history of childhood abuse.

**Conclusions and relevance:**

In this explorative cross-sectional study, strong attachment to pets, especially dogs, was associated with lower anxiety and depression symptoms. The favorable association was particularly apparent in women with a history of childhood abuse.

## Key Points

**Question:**

Is bonding closely with a pet associated with reduced depression and anxiety, especially for vulnerable groups like adults who experienced childhood abuse?

**Findings:**

In this cross-sectional study including 214 women with an oversampling of childhood abuse survivors (156 participants [72.6%]), higher pet attachment was associated with lower generalized anxiety. Dog attachment was associated with reduced depression, anxiety, and overall anxiety and depression symptoms, especially among abuse survivors.

**Meaning:**

These results suggest that strong bonds with pets, especially dogs, may alleviate anxiety and depression, aiding mental health interventions, particularly for vulnerable groups.

## Teaser

This cohort study examines the association of dog and cat attachment with depression and anxiety scores in middle-aged and older women, in particular those who reported experiencing abuse as children.

## Introduction

Depression and anxiety are common among women. In recent data among midlife (aged 45 to 64 years) and older (aged 65 years or older) US adults, more women (21.8%) than men (15.0%) experienced symptoms of depression in the past 2 weeks.^1^ Similarly, anxiety symptoms experienced in the past 2 weeks were reported by 15.2% of midlife and 11.2% of older adults, with women (19%) again faring worse than men (11.9%).^2^ Various modifiable social, behavioral, and health determinants, including by gender, may influence this high burden of depression and anxiety.^3,4,5,6,7,8^ Therefore, means for prevention of depression and anxiety, potentially through targeting of modifiable factors, need to be identified—particularly among high-risk subpopulations.

Depression and anxiety among pet owners has been studied previously among midlife and older adult populations^9,10,11,12,13,14,15,16^; however, findings have been mixed, likely due to methodologic issues such as self-selection into pet ownership, unmeasured confounding,^17,18^ and limited consideration of reverse causality.^19,20^ Furthermore, it is plausible that certain subpopulations at higher risk of depression and/or anxiety, such as adolescents who are mistreated by peers,^21^ LGBTQ+ emerging adults (ages 18 to 29 years),^22^ adolescents who experienced childhood abuse,^23^ or older adults facing social loss^24^ may benefit from pet ownership. Moreover, preliminary evidence among some groups at higher risk for depression and anxiety,^25,26^ such as people living with HIV/AIDS^27^ or terminal cancer,^28^ has shown an inverse association between active engagement with pets and levels of depression and anxiety. Such findings suggest that these potentially protective associations may be more attributable to levels of engagement with and attachment to pets, rather than simple pet ownership itself. In some prior work, higher pet attachment was associated with higher depression scores^29,30,31^; however, as shown by studies addressing mediation,^32^ this positive association appears to be primarily driven by the nature of the human attachment style, where stronger pet attachment is positively associated with insecure human attachment style and negatively associated with secure human attachment style.

Experiencing childhood abuse is associated with increased risk of depression and anxiety^33^ over the life course, and can affect human attachment style^34^ used as a pattern throughout life.^35^ In the current study, we focused on a group of women at elevated risk for depression and anxiety: participants in the Mind Body Study (MBS),^36^ a cohort in which women with a history of physical or sexual childhood abuse were randomly oversampled to obtain a group with increased likelihood of having psychosocial distress. In this MBS sample we examined the association between pet attachment and depression and anxiety, hypothesizing that higher levels of pet attachment may be protective against depression and anxiety symptoms. We further hypothesized that, among women with a history of childhood abuse, greater pet attachment may be particularly protective against depression and anxiety symptoms, potentially buffering negative prior social experiences with people.^32^

## Methods

### Mind Body Study

In 2013, 688 participants in the Nurses’ Health Study II (NHS2)—a longitudinal cohort of registered US nurses—were invited to take part in the MBS with a primary motivation to investigate how psychosocial factors may affect biomarkers associated with cancer progression. Accordingly, women were invited to participate in the MBS who had provided blood and urine samples in an earlier study (1996-1999, 29 611 participants) and in one of the later follow-up studies collecting biological samples.^36^ To increase the proportion of individuals with psychosocial suffering in adulthood, women who previously reported experiencing childhood abuse were randomly oversampled (33% in MBS vs 14% in NHS2).^36^ Compared with NHS2 women, MBS participants were more likely to be on psychotropic medications but less likely to experience chronic conditions such as diabetes and hypertension.^36^

The study protocol was approved by the institutional review board of Brigham and Women’s Hospital and the Committee on the Use of Human Subjects in Research of Harvard T.H. Chan School of Public Health. Voluntary return of questionnaires indicated informed consent. This report follows the Strengthening the Reporting of Observational Studies in Epidemiology (STROBE) reporting guideline for cross-sectional studies.

### Assessment of Pet Attachment

In the 2 online surveys, participants were asked, “Do you currently have one or more of the following pets in the household? Please choose only one of the following: dog, cat, bird, fish, other pets, no pets in the household.” Those who declared owning any pets were also asked 6 questions from the Lexington Attachment to Pets Scale (LAPS)^37^ that were supposed to refer to the declared pet type. LAPS questions assessed owners’ feelings and behaviors toward the pet they spend the most time with, such as considering them a friend, talking to them, finding happiness in owning a pet, discussing their pet with others, playing with and considering their pet as a part of the family. Participants could respond with 5 options—strongly disagree, disagree, agree, strongly agree, or do not know—which were assigned numerical values. The total LAPS score was calculated by summing up the responses, with higher scores indicating higher attachment.

We considered as exposed those who provided LAPS assessments at least once, and as their measure of exposure we used the LAPS averaged over available assessments. We assumed that LAPS refers to a dog if they declared owning a dog twice or a dog once and an animal other than a cat or no animal once. Similarly, we assumed that LAPS refers to a cat if they declared owning a cat twice or a cat once and an animal other than a dog or no animal once. We were unable to determine which species the LAPS refers to if they declared owning a dog once and owning a cat once, they declared owning a pet other than dog or cat twice, or they declared owning a pet other than dog or cat once and not answered to the pet ownership question in the other assessment (Table 1).

**Table 1.** Definition of Exposure Groups and Corresponding LAPS Attachment Groups for MBS Study Participants

Answer to pet ownership question		Assigned exposure group	LAPS attachment group
Survey 1	Survey 2		
No pets in household	No pets in household	Unexposed (no pet)	No LAPS assessed
No pets in household	Missing or no return		
Missing or no return	No pets in household		
Dog	Dog	Exposed (dog)	Dog attachment
Dog	Bird, fish, other pet,^a^ missing, or no return		
Bird, fish, other pet, missing, or no return	Dog		
Cat	Cat	Exposed (cat)	Cat attachment
Cat	Bird, fish, other pet,^a^ missing, or no return		
Bird, fish, other pet,^a^ missing. or no return	Cat		
Cat	Dog	Exposed (mixed)	Not able to determine
Dog	Cat		
Bird or fish or other pet^a^	No pets in household		

Abbreviations: LAPS, Lexington Attachment to Pets Scale; MBS, Mind Body Study.

^a^ Does not include dogs or cats.

### Depression and Anxiety Outcomes

Depression symptoms were measured using the 10-item Center for Epidemiologic Studies–Depression scale (CESD-10)^38^ and Kessler Psychological Distress Scale (K6).^39^ Anxiety symptoms were measured using the Crown Crisp Experiential Index (CCI)^40^ and the Generalized Anxiety Disorder-7 (GAD-7)^41^ scale. The MBS featured repeated questionnaires to facilitate testing of the reliability of these measures^36^; thus, in our primary analysis we used the averaged values over available assessments. Mean CESD-10, K6, CCI, and GAD-7 scale scores were analyzed as continuous outcomes. In addition, we generated an overall global measure of depression and anxiety, by first *z*-scoring each of the 4 scales, and then averaging the 4 resulting *z*-scores.

In secondary analyses, we used measure-specific cut-off points to define dichotomous outcomes indicating the presence or absence of clinically important depression or anxiety symptoms. Clinically relevant depressive symptoms are defined by scores of 10 or higher for CESD-10^42^ and 12 or higher for the K6.^43^ For CCI, a cut-off of 6 points was previously validated for defining clinically relevant anxiety symptoms.^44,45^ For GAD-7, a cut-off of 5 points identifies probable GAD.^46,47^ These established cutoffs were applied to the corresponding continuous mean measures, defining their dichotomized versions. We also defined a composite binary outcome variable (yes or no) as having clinically important symptoms of depression and/or anxiety on any of the scales.

### Covariates

We considered age, body mass index (BMI; calculated as weight in kilograms divided by height in meters squared), marital status (married, not married), number of stressful events during past 6 months, presence of life-threatening events (anytime in lifetime), history of physical and/or sexual childhood abuse, alcohol consumption (in grams per day), physical activity (in metabolic equivalent task [MET] hours/week), and the Berkman–Syme Social Network Index (BSSNI; measure of social integration)^48^ as potential confounders. Freiburg Mindfulness Inventory (FMI),^49^ a general measure of mindfulness, was also included in our models as a factor potentially associated with pet attachment and depression and anxiety. Except for a history of physical or sexual childhood abuse assessed in 2001, and height from 1989 used to calculate BMI, all other covariates were assessed concurrently in both MBS online surveys. As we did for the outcome measures, we averaged values of the continuous covariates over their available assessments. For stressful life events we assigned a positive response if reported in either of the 2 surveys.

### Modeling Strategy

For each of the 5 continuous outcome variables we used multivariable generalized linear models with averaged continuous LAPS as the exposure. We considered models adjusted for (1) age^1,2,3,4,50,51,52^ and (2) age, BMI,^53,54,55,56^ marital status,^57,58,59,60^ number of stressful events during past 6 months, presence of life-threatening events, history of physical and/or sexual childhood abuse,^61,62,63,64^ alcohol consumption,^65,66^ physical activity,^67,68,69,70^ BSSNI,^53,59,71,72^ and FMI.^73,74,75,76,77,78^ We performed analogous analyses for the 4 defined dichotomous outcomes using logistic regression models. In addition, we evaluated whether results differed when restricting to women with childhood abuse experience, those who were married (about 75% of the study sample) and when stratifying by pet type (dog, cat). To preserve the familywise error rate (FWER) at the level of 0.05, we used Bonferroni correction for multiple testing leading to the significance level for an individual test of .005. All analyses were conducted using SAS software, version 9.4 (SAS Institute, Inc).

## Results

Our study sample consisted of 214 MBS participants who answered the question on pet ownership at least once (mean [SD] age, 60.8 [3.9] years). Of the 688 women invited, 293 (42.6%) expressed interest and received the online questionnaire twice (March 2013 and February 2014), with response rates exceeding 70% (2013 questionnaire, 228 participants [77.8%]; 2014 questionnaire, 208 participants [71.0%]).^36^ Compared with all invited, responders were more likely to be White, had lower BMI, were less likely to have experienced childhood abuse and less likely experienced hypertension and type 2 diabetes. The exposed group consisted of 140 individuals who provided at least 1 LAPS assessment. Seventy-four women declared not owning a pet at least once, constituting our reference group of the unexposed. The averaged LAPS measure was strongly left-skewed in our study sample (Table 2), indicating high attachment.

**Table 2.** Characteristics of MBS Study Participants Who Reported Pet Ownership at Least Once by Pet Type

Characteristic	Participants, No. (%)					
	Total (n = 214)	No pet (n = 74)	Dog (n = 78)	Cat (n = 46)	Mixed (n = 15)	Missing, No. (%)
LAPS,^a^ median (IQR) [range]	17 (13-18) [0-18]	NA	17 (13.5-18) [7-18]	16 (13-18) [0-18]	16.5 (8.5-18) [4-18]	0^a^
Continuous outcomes^b^ median (IQR)						
CES-D-10	5.0 (2.2-8)	4.5 (2.0-7.0)	5.0 (2.0-7.5)	6.0 (2.5-8.5)	6.5 (3.5-10.0)	0
CCI	3.0 (2.0-4.5)	2.5 (1.5-4.5)	3.0 (2.0-4.5)	3.0 (2.0-5.0)	3.5 (2.0-4.0)	5 (2.3)
GAD-7	1.5 (0.5-4.0)	1.0 (0-4.0)	1.5 (0.5-3.5)	2.8 (0.5-4.0)	2.0 (1.0-3.5)	0
K6	2.0 (1.0-3.5)	2.0 (0.5-3.0)	2.0 (0.5-3.5)	2.5 (1.0-4.5)	2.0 (1.0-4.0)	0
*z*-score	−0.2 (−0.6 to 0.4)	−0.2 (−0.6 to 0.2)	−0.2 (−0.6 to 0.3)	−0.02 (−0.4 to 0.6)	0.2 (−0.4 to 0.6)	0
Dichotomized outcomes						
CES-D-10 ≥10	32 (15.0)	13 (17.6)	7 (9.0)	8 (17.4)	4 (26.7)	0
CCI ≥6	24 (11.5)	5 (6.9)	10 (13.3)	8 (17.8)	1 (6.7)	5 (2.3)
GAD-7 ≥5	38 (17.8)	17 (23.0)	10 (12.8)	9 (19.6)	2 (13.3)	0
Any clinically important depression or anxiety symptoms	62 (29.0)	21 (28.4)	19 (24.4)	17 (37.0)	5 (33.3)	0
Demographic and psychosocial characteristics						
Age, mean (SD), y	60.8 (3.9)	61.3 (3.6)	60.1 (3.9)	61 (4.1)	61.3 (4.2)	0
BMI, mean (SD)^c^	26.4 (5.3)	25.2 (3)	26.9 (3.7)	26.7 (3.7)	28.5 (2.6)	0
Married^c^	160 (74.8)	53 (72.9)	64 (84.1)	31 (65.5)	12 (80.0)	0
Pittsburgh Sleep Quality Index, mean (SD)^c^	0.9 (0.4)	0.9 (0.3)	1 (0.3)	1 (0.3)	1 (0.1)	0
Stressful events during past 6 mos, mean (SD), No.^c^	1.2 (1.3)	1.1 (0.8)	1.3 (1.1)	1.1 (0.6)	1.6 (0.7)	3 (1.4)
Life-threatening events, ever in life^c^	56 (26.3)	20 (29.4)	17 (23.4)	18 (37.4)	1 (6.7)	1 (0.5)
Mindful Attention Awareness Scale, mean (SD)^c^	23.8 (3.5)	24.6 (2.9)	23.5 (2.8)	23.6 (2)	24.2 (1.1)	0
Freiburg mindfulness inventory, mean (SD)^c^	14.8 (2.5)	14.9 (2)	14.8 (1.9)	14.7 (1.5)	15.1 (0.9)	0
Physical/sexual abuse^c^	151 (72.6)	52 (70.8)	58 (77.6)	29 (63.5)	12 (83.3)	6 (2.8)
Alcohol consumption, mean (SD), g/d^c^^,^^d^	7.6 (11)	8.5 (8.2)	9.5 (10.5)	5.7 (5.2)	9.2 (4)	0
Physical activity, mean (SD), MET h/wk^c^	30.6 (28.9)	35.4 (22.9)	27 (21.2)	24.4 (16)	34.4 (11.8)	0
Berkman social network index						0
Fully socially isolated	12 (5.6)	4 (5.4)	6 (7.7)	2 (4.4)	0	
Moderately isolated	40 (18.7)	14 (18.9)	15 (19.2)	6 (13.0)	5 (33.3)	
Moderately integrated	88 (41.1)	30 (40.5)	28 (35.9)	23 (50.0)	6 (40.0)	
Fully integrated	74 (34.6)	26 (35.1)	29 (37.2)	15 (32.6)	4 (26.7)	

Abbreviations: CCI, Crown Crisp Experiential Index phobic anxiety subscale; CES-D-10, 10-item Centre for Epidemiologic Studies Depression Scale; GAD-7, 7-item Generalized Anxiety Disorder; K6, Kessler Psychological Distress Scale; LAPS, Lexington Attachment to Pets Scale; MET, metabolic equivalent tasks; NA, not applicable.

^a^ Among 140 pet owners.

^b^ Score ranges for continuous outcomes: CES-D-10 (0-30); CCI (0-16); GAD-7 (0-21); K6 (0-24).

^c^ Value is standardized to the age distribution of the study population.

^d^ Assessed in 2011.

Compared with NHS2 participants, women in our sample were older, had lower BMI and were more likely to have experienced childhood abuse (Table 2; eTable 1 in Supplement 1; Huang et al^36^). Compared with participants with dog attachments, those attached to cats, on average, were more likely to have experienced life-threatening events (18 [37.4%] vs 17 [23.4%]), and less likely to have experienced childhood abuse (29 [63.5%] vs 58 [77.6%]). Participants with cat attachments had also higher median (IQR) measures of depression (CES-D-10: 6.0 [2.5-8.5] vs 5.0 [2.0-7.5]) and anxiety (GAD-7: 2.8 [0.5-4.0] vs 1.5 [0.5-3.5]) and were more likely than individuals with dog attachments to experience clinically significant symptoms of depression or anxiety (17 of 46 [37.0%] vs 19 of 78 [24.4%]) (Table 2).

Overall, higher pet attachment was associated with lower depression and anxiety symptoms, except CCI, although only the associations with the GAD-7 (β = −0.17; 95% CI, −0.29 to −0.06; *P* = .004) were statistically significant (Table 3). Higher dog attachment was associated with lower mean CESD-10 depression scores (β = −0.47; 95% CI, −0.68 to −0.26; *P* < .001), lower mean K6 distress scores (β = −0.42; 95% CI, −0.54 to −0.31; *P* < .001), lower mean GAD-7 scores (β = −0.47; 95% CI, −0.65 to −0.30; *P* < .001), as well as lower overall *z*-score for anxiety and depression (β = −0.12; 95% CI, −0.17 to −0.08; *P* < .001). None of the variables included in the multivariable model had significant mediating effects in the group of people attached to the dog for any of the outcome variables considered. There were no significant associations between cat-attachment levels and depression and anxiety scores and there was little evidence of associations in the mixed group (Table 3). Analysis of dichotomized outcomes (ie, for relations of pet attachment to likelihood of being above vs below prespecified clinical cut-off for depression or anxiety) showed null associations with limited power (eTable 1 in Supplement 1). Restricting analyses to those with histories of childhood abuse reduced our sample to 156 women (57 non–pet owners; 99 with pet attachments; 58 dog attachments and 29 with cat attachments). Overall, we observed stronger point estimates for associations between pet attachment and CESD-10, GAD-7, and K6 scores (Table 4). In this high-risk subset, results were similar to those from the main analyses among participants with dog attachments (CESD-10: β = −0.47; 95% CI, −0.69 to −0.24; K6: β, −0.43; 95% CI, −0.59 to −0.34; GAD-7: β = −0.5; 95% CI, −0.70 to −0.30; *z*-score: β = −0.12; 95% CI, −0.17 to −0.08), whereas associations between cat attachment and depression and anxiety scores remained nonsignificant (Table 4). Interaction between childhood abuse and LAPS was not significant (Table 5). Also, analyses in a group of married women showed no significant differences from overall results (eTables 2 and 3 in Supplement 1).

**Table 3.** LAPS and Mean Scores of Depression and Anxiety Among MBS Participants (N = 140)

Measure	Model^a^	Pet			Dog			Cat			Mixed		
		Total, No.	LAPS β (95% CI)	*P* value	Total, No.	LAPS β (95% CI)	*P* value	Total, No.	LAPS β (95% CI)	*P* value	Total, No.	LAPS β (95% CI)	*P* value
CESD-10	1	140	−0.04 (−0.22 to 0.14)	.67	78	−0.27 (−0.52 to −0.03)	.03	46	0.27 (−0.08 to 0.62)	.14	15	0.11 (−0.34 to 0.55)	.64
	2	134	−0.16 (−0.32 to −0.01)	.04	75	−0.47 (−0.68 to −0.26)	<.001	44	0.14 (−0.25 to 0.44)	.59	14	−0.15 (−0.45 to 0.14)	.31
K6	1	140	−0.05 (−0.15 to 0.06)	.36	78	−0.26 (−0.4 to −0.12)	<.001	46	0.13(−0.09 to 0.35)	.24	15	0.06 (−0.16 to 0.28)	.60
	2	134	−0.12 (−0.21 to −0.03)	.01	75	−0.42 (−0.54 to −0.31)	<.001	44	0.09 (−0.11 to 0.28)	.38	14	0.04 (−0.03 to 0.11)	.23
CCI	1	136	0.02 (−0.08 to 0.12)	.64	75	0.01 (−0.13 to 0.16)	.87	45	0.07 (−0.15 to 0.3)	.51	15	−0.06 (−0.19 to 0.08)	.43
	2	130	0.01 (−0.09 to 0.12)	.80	72	−0.08 (−0.24 to 0.09)	.36	43	0.08 (−0.17 to 0.33)	.54	14	0 (−0.05 to 0.04)	.89
GAD-7	1	140	−0.09 (−0.22 to 0.04)	.19	78	−0.32 (−0.51 to −0.13)	.001	46	0.19 (−0.07 to 0.46)	.15	15	−0.06 (−0.3 to 0.18)	.65
	2	134	−0.17 (−0.29 to −0.06)	.004	75	−0.47 (−0.65 to −0.3)	<.001	44	0.17 (−0.04 to 0.37)	.12	14	−0.17 (−0.25 to −0.09)	<.001
*z*-score	1	140	−0.01 (−0.05 to 0.02)	.46	78	−0.07 (−0.12 to −0.02)	.005	46	0.06 (−0.02 to 0.13)	.14	15	0 (−0.06 to 0.06)	.98
	2	134	−0.04 (−0.07 to −0.01)	.01	75	−0.12 (−0.17 to −0.08)	<.001	44	0.04 (−0.03 to 0.1)	.25	14	−0.02 (−0.04 to 0.00)	.05

Abbreviations: BMI, body mass index (calculated as weight in kilograms divided by height in meters squared); BSSI, Berkman-Syme social index; FMI, Freiburg Mindfulness Inventory; GAD-7, 7-item Generalized Anxiety Disorder; LAPS, Lexington Attachment to Pets Scale; MET, metabolic equivalent tasks.

^a^ Model 1 was adjusted for age and LAPS. Model 2 was adjusted for LAPS, age, BMI, marital status number of stressful events during past 6 months, a presence of life-threatening events (anytime in lifetime), history of physical or sexual abuse, FMI, alcohol consumption, physical activity (METs), and BSSI.

**Table 4.** Mean LAPS and Scores of Depression and Anxiety Among MBS Participants (N = 99)

Measure	Model^a^	Pet			Dog			Cat			Mixed		
		Total, No.	LAPS β (95% CI)^b^	*P* value	Total, No.	LAPS β (95% CI)^b^	*P* value	Total, No.	LAPS β (95% CI)^b^	*P* value	Total, No.	LAPS β (95% CI)^b^	*P* value
CES-D-10	1	99	−0.16 (−0.41 to 0.09)	.21	58	−0.4 (−0.69 to −0.11)	.01	29	0.53 (−0.2 to 1.26)	.16	12	0.04 (−0.49 to 0.57)	.88
	2	99	−0.25 (−0.43 to −0.07)	.01	58	−0.47 (−0.69 to −0.24)	<.001	29	0.26 (−0.23 to 0.75)	.29	12	−0.04 (−0.64 to 0.55)	.89
K6	1	99	−0.11 (−0.26 to 0.03)	.12	58	−0.35 (−0.52 to −0.18)	<.001	29	0.33 (−0.09 to 0.75)	.12	12	0.06 (−0.17 to 0.29)	.60
	2	99	−0.17 (−0.28 to −0.06)	.003	58	−0.47 (−0.59 to −0.34)	<.001	29	0.11 (−0.17 to 0.39)	.45	12	0.07 (−0.06 to 0.2)	.30
CCI	1	95	0.05 (−0.09 to 0.19)	.50	55	0.06 (−0.12 to 0.24)	.52	28	0.26 (−0.23 to 0.75)	.30	12	−0.07 (−0.24 to 0.1)	.42
	2	95	0.04 (−0.1 to 0.17)	.59	55	−0.01 (−0.18 to 0.16)	.91	28	−0.04 (−0.45 to 0.36)	.83	12	0.1 (0.04 to 0.16)	.001
GAD-7	1	99	−0.18 (−0.36 to 0)	.05	58	−0.43 (−0.67 to −0.19)	.003	29	0.51 (0.07 to 0.96)	.02	12	−0.08 (−0.3 to 0.13)	.44
	2	99	−0.22 (−0.37 to −0.07)	.004	58	−0.5 (−0.7 to −0.3)	.004	29	0.27 (−0.05 to 0.59)	.10	12	−0.39 (−0.43 to −0.35)	<.001
Z-score	1	99	−0.03 (−0.08 to 0.02)	.19	58	−0.09 (−0.15 to −0.03)	.003	29	0.14 (−0.01 to 0.28)	.06	12	−0.01 (−0.07 to 0.05)	.82
	2	99	−0.05 (−0.09 to −0.01)	.01	58	−0.12 (−0.17 to −0.08)	<.001	29	0.04 (−0.04 to 0.12)	.34	12	−0.02 (−0.06 to 0.02)	.45

Abbreviations: BMI, body mass index (calculated as weight in kilograms divided by height in meters squared); BSSI, Berkman-Syme social index; FMI, Freiburg Mindfulness Inventory; GAD-7, 7-item Generalized Anxiety Disorder; LAPS, Lexington Attachment to Pets Scale; MET, metabolic equivalent tasks.

^a^ Model 1 was adjusted for age and LAPS. Model 2 was adjusted for LAPS, age, BMI, marital status number of stressful events during past 6 months, a presence of life-threatening events (anytime in lifetime), FMI, alcohol consumption, physical activity (METs), and BSSI.

^b^ β coefficients are effect estimates from linear regression models.

**Table 5.** *P* Values for Interaction Between Mean LAPS and Childhood Abuse in Models^a^ for Mean Scores of Depression and Anxiety Among MBS Participants (N = 140)

Outcome	Pet	Dog	Cat
CES-D-10	.8050	.6492	.2582
K6	.5976	.0554	.3986
CCI	.9134	.8772	.8473
GAD-7	.9233	.5551	.5165
*z*-score	.9662	.3343	.4121

Abbreviations: BMI, body mass index (calculated as weight in kilograms divided by height in meters squared); BSSI, Berkman-Syme social index; FMI, Freiburg Mindfulness Inventory; GAD-7, 7-item Generalized Anxiety Disorder; LAPS, Lexington Attachment to Pets Scale; MET, metabolic equivalent tasks.

^a^ Models contained LAPS, childhood abuse, LAPS × childhood abuse, age, BMI, marital status number of stressful events during past 6 months, a presence of life-threatening events (anytime in lifetime), FMI, alcohol consumption, physical activity (METs), and BSSI.

## Discussion

In this innovative study, we found that higher attachment to pets, especially dogs, was significantly associated with lower anxiety and depression symptoms. Estimates for these associations appeared even stronger among women with histories of childhood physical and/or sexual abuse.

These findings may be considered in the context of previous attachment research. Bowlby’s attachment theory^35,79^ states that the interaction with the primary attachment figure establishes the attachment style (secure or insecure^80,81^), which becomes an internal working model and can be transferred to other close relationships later in life.^35^ A secure attachment style in adults has been found to be associated with better mental health,^82,83,84^ whereas insecure attachment style is associated with higher risk of depression and anxiety.^85^ Studies on pet attachment have shown that attachment theory may be extended to human-animal attachment,^86^ where the pet acts as an attachment figure. Of note, there is evidence that people with insecure human attachment styles tend to develop strong, secure pet attachment,^87^ potentially as a compensatory strategy substituting secure human attachment.^32^

We examined the association between pet attachment and depression and anxiety in a group with overrepresentation of women with experience of childhood abuse, which has been found to be associated with an increased risk of depression,^88,89^ anxiety,^33^ and other psychopathology^90^ in adulthood. Childhood abuse also increases the chances of developing an insecure human-attachment style,^34,91^ and the pet-attachment level among women with childhood abuse has been shown to be higher than among women without childhood abuse experience.^92^

In our sample, we observed statistically significant associations between higher pet attachment and lower generalized anxiety symptoms overall, and strong correspondence between higher pet attachment and lower symptoms of both depression and anxiety among dog owners in particular. Although statistically significant, these results cannot be interpreted as clinically meaningful, due to effect sizes lower than established minimal clinically important differences^103^ or smallest detectable change,^104^ or due to lack of established minimal clinically important differences.^105^ However, obtained effects are in line with previous research findings^106,107^ and regardless of clinical significance, reductions in psychological distress are associated with better overall mental health.^108,109^

The majority of prior studies dealing with the relationship between pet attachment and the owner’s depression and or anxiety outcomes suggested that high attachment levels were associated with poorer mental health.^29,30,31,93,94,95^ Other studies, including the current study, that found the opposite association (ie, higher pet attachment and better mental health outcomes) had considered specific subpopulations (eg, individuals identifying as LGBTQ+,^31^ employees with unsecure job conditions during COVID-19,^96^ people with low levels of resilience and exposed to COVID-19 restrictions,^97^ people with moderate to high levels of poor mental health symptoms^98^) that were at increased risk of depression and anxiety. In this context, our results expand the evidence base to address another at-risk group: women with histories of childhood physical and/or sexual abuse. In addition, our results are in keeping with results from a recent mediation analysis,^32^ in which a key factor shaping the relationship between levels of pet attachment and depression and anxiety appeared to be the internal working model for building human attachments acquired in childhood. Thus, while this remains speculative, it is possible that among people with insecure human attachment styles related to childhood abuse, pet attachment may play a special role by compensating for the lack of secure relationships with people and thereby protecting against depression and anxiety symptoms.

### Strengths and Limitations

A major strength of our analysis is the well-characterized MBS sample, which was enriched for psychosocial and stress variables and afforded the opportunity to analyze the complex human-animal relationship in a unique group with an overrepresentation of women with experience of childhood abuse—a group among whom building a bond with the animal may proceed differently than in the general population and may be of particular importance to the pet owner.

This study also has limitations. Our results were statistically significant only in the group of participants who had formed dog attachments and not among participants with cat attachments, which may be due to small sample size (46 cat vs 78 dog), differences in demographic, psychosocial, and personality-specific factors as well as other, unmeasured factors between dog and cat owners. Further research in this group is needed to make inferences about the relationship between cat attachment and depression and anxiety. Another limitation is the cross-sectional nature of our analyses and thus potential for reverse causality. However, in contrast to the acquisition of a pet, which may exhibit a higher susceptibility to reverse causation, such as individuals seeking companionship to mitigate loneliness or mitigate depressive symptoms, it is considerably less probable that attachment to one’s pet would intensify as a consequence of mental health conditions. Rather, research findings indicate that attachment insecurity plays a significant role in the development of mental disorders, and that strengthening attachment security can help improve psychopathological symptoms.^99^ A 2022 meta-analysis^100^ came to the conclusion that adult attachment was significantly associated with 10 different mental health outcomes in the overwhelming majority of the 224 studies identified. Moderating variables of note were age, gender, marital status, ethnicity, and study type (cross-sectional vs longitudinal studies). However, contrary to prior research that had suggested that the relationship between adult attachment and mental health outcomes may differ between cross-sectional and longitudinal studies,^101,102^ study type (cross-sectional and longitudinal) was not identified as a moderator of the link between adult attachment and mental health in the meta-analysis by Zhang et al.^100^ In fact, only gender was significantly moderating these effects. Our study of middle-aged to older, mostly white women, the majority of whom were married, addresses these concerns. Lastly, that we see starkly diverging results between dog and cat attachment may further limit concerns regarding reverse causality, as it may appear even less likely that only dog attachment, but not cat attachment, would increase as a result of depressive symptoms (under the reverse causality theory). Nonetheless, the characteristics of the specific group of women (nurses) in this study sample may limit generalizability to the broader population of those affected by childhood abuse, including men—highlighting the need for further longitudinal studies among people with childhood abuse.

## Conclusions

Results from this cross-sectional study suggest there may be protective associations between higher pet attachment, and dog attachment in particular, and lower levels of depression and anxiety symptoms among women—especially among women with histories of adverse childhood experiences of physical and/or sexual abuse. While not directly applicable to clinical practice, our results point to an important aspect of pet attachment as a factor improving the psychological well-being of particularly vulnerable owners, and will therefore also be of value to clinicians. Moreover, the results add novel findings to research regarding the complex nature and potential consequences of the human-animal bond among those who may have developed an insecure human attachment style due to childhood trauma. This knowledge also informs the scientific premise for further novel work addressing pet attachment as a means to develop preventive and therapeutic strategies for depression and anxiety in this vulnerable group of people.

## Notes

Supplement 1. **eTable 1.** Association Between Mean Lexington Attachment to Pet Score (LAPS) and Dichotomized Averaged Scores of Depressions (CESD10), Anxiety (CCI, GAD7) and Any Clinically Important Depression or Anxiety Symptoms Among Mind Body Study Participants Who at Least Once Reported on Pet Ownership and LAPS **eTable 2.** Association Between Mean Lexington Attachment to Pet Score (LAPS) and Averaged Scores of Depression (CESD10, K6) and Anxiety (CCI, GAD7) Among Mind Body Study Participants Who at Least Once Reported on Pet Ownership and LAPS and Were Married **eTable 3.** Association Between Mean Lexington Attachment to Pet Score (LAPS) and Averaged Scores of Depression (CESD10, K6) and Anxiety (CCI, GAD7) Among Mind Body Study Participants Who at Least Once Reported on Pet Ownership and LAPS and Were Not Married

Supplement 2.
Data Sharing Statement
