# Supplementary material for: Pet Attachment and Anxiety and Depression in Middle-Aged and Older Women
Source: JAMA Netw Open. 2024 Aug 1;7(8):e2424810. doi: 10.1001/jamanetworkopen.2024.24810 (PMC11294964; doi:10.1001/jamanetworkopen.2024.24810)
Supplement: Supplement 1. — eTable 1. Association Between Mean Lexington Attachment to Pet Score (LAPS) and Dichotomized Averaged Scores of Depressions (CESD10), Anxiety (CCI, GAD7) and Any Clinically Important Depression or Anxiety Symptoms Among Mind Body Study Participants Who at Least Once Reported on Pet Ownership and LAPS eTable 2. Association Between Mean Lexington Attachment to Pet Score (LAPS) and Averaged Scores of Depression (CESD10, K6) and Anxiety (CCI, GAD7) Among Mind Body Study Participants Who at Least Once Reported on Pet Ownership and LAPS and Were Married eTable 3. Association Between Mean Lexington Attachment to Pet Score (LAPS) and Averaged Scores of Depression (CESD10, K6) and Anxiety (CCI, GAD7) Among Mind Body Study Participants Who at Least Once Reported on Pet Ownership and LAPS and Were Not Married [file jamanetwopen-e2424810-s001.pdf]

Supplemental Online Content

Żebrowska M, Strohmaier S, Huttenhower C, et al. Pet Attachment and Anxiety and Depression in Middle-Aged and Older Women. *JAMA Netw Open.* 2024;7(7):e2424810. doi:10.1001/jamanetworkopen.2024.24810

eTable 1. Association Between Mean Lexington Attachment to Pet Score (LAPS) and Dichotomized Averaged Scores of Depressions (CESD10), Anxiety (CCI, GAD7) and Any Clinically Important Depression or Anxiety Symptoms Among Mind Body Study Participants Who at Least Once Reported on Pet Ownership and LAPS

eTable 2. Association Between Mean Lexington Attachment to Pet Score (LAPS) and Averaged Scores of Depression (CESD10, K6) and Anxiety (CCI, GAD7) Among Mind Body Study Participants Who at Least Once Reported on Pet Ownership and LAPS and Were Married

eTable 3. Association Between Mean Lexington Attachment to Pet Score (LAPS) and Averaged Scores of Depression (CESD10, K6) and Anxiety (CCI, GAD7) Among Mind Body Study Participants Who at Least Once Reported on Pet Ownership and LAPS and Were Not Married

This supplemental material has been provided by the authors to give readers additional information about their work.

© 2024 Żebrowska M et al. *JAMA Network Open*.

eTable 1. Association between mean Lexington attachment to pet score (LAPS) and dichotomized averaged scores of depression (CESD10), anxiety (CCI, GAD7) and any clinically important depression or anxiety symptoms among Mind Body Study participants who at least once reported on pet ownership and LAPS (N=140). Effect estimates are odds ratios (OR) and 95% confidence intervals (95%CI).

Dichotomous outcome	Model	Pet		Dog		Cat	
		N(cases)	OR (95%CI)	N(cases)	OR (95%CI)	N(cases)	OR (95%CI)
CESD10 ≥10	Age adj.	140(19)	0.96(0.85 to 1.1)	78(7)	0.81(0.65 to 1.0)	46(8)	1.02(0.84 to 1.38)
	MV adj.	134(19)	0.87(0.74 to 1.02)	75(7)	--*	44(8)	1.02(0.66 to 1.45)
CCI ≥ 6	Age adj.	136(19)	1.02(0.9 to 1.2)	75(10)	1.03(0.84 to 1.33)	45(8)	1.06(0.86 to 1.5)
	MV adj.	130(19)	1.01(0.87 to 1.21)	72(10)	0.98(0.78 to 1.26)	43(8)	--
GAD-7 ≥5	Age adj.	140(21)	0.97(0.86 to 1.11)	78(10)	0.85(0.7 to 1.02)	46(9)	1.12(0.89 to 1.57)
	MV adj.	134(20)	0.94(0.82 to 1.08)	75(10)	0.84(0.66 to 1.05)	44(8)	1.06(0.63 to 1.6)
Any clinically important depression or anxiety symptoms	Age adj.	140(41)	1.0(0.91 to 1.11)	78(19)	0.93(0.8 to 1.1)	46(17)	1.06(0.89 to 1.34)
	MV adj.	134(40)	0.95(0.84 to 1.07)	75(19)	0.85(0.65 to 1.05)	44(16)	0.94(0.7 to 1.29)

Age adj.: models containing LAPS and age. MV adj.: models containing LAPS, age, body mass index (BMI), marital status number of stressful events during past 6 months, a presence of life-threatening events (anytime in lifetime), Freiburg Mindfulness Inventory (FMI), alcohol consumption, physical activity (METs) and Berkman-Syme social index (BSSI). * Model did not converge.

© 2024 Żebrowska M et al. *JAMA Network Open*.

eTable 2. Association between mean Lexington attachment to pet score (LAPS) and averaged scores of depression (CESD10, K6) and anxiety (CCI, GAD7) among Mind Body Study participants who at least once reported on pet ownership and LAPS and **were married**. β_{LAPS} are effect estimates from linear regression models.

		**Pet**			**Dog**			**Cat**			**Mixed**		
	**Model**	**N**	**β_{LAPS} (95%CI)**	**p**	**N**	**β_{LAPS} (95%CI)**	**p**	**N**	**β_{LAPS} (95%CI)**	**p value**	**N**	**β_{LAPS} (95%CI)**	**p**
CES-D-10	Age adj.	107	-0.05 (-0.25 to 0.14)	0.59	64	-0.18 (-0.45 to 0.08)	0.17	31	0.12 (-0.25 to 0.49)	0.53	12	0.1 (-0.47 to 0.67)	0.73
	MV adj.	101	-0.15 (-0.32 to 0.01)	0.07	60	-0.34 (-0.58 to -0.09)	0.01	30	-0.03 (-0.42 to 0.36)	0.87	11	-*	-
K6	Age adj.	107	-0.05 (-0.16 to 0.07)	0.41	64	-0.22 (-0.36 to -0.07)	<0.01	31	0.12 (-0.13 to 0.36)	0.35	12	0.03 (-0.24 to 0.31)	0.80
	MV adj.	101	-0.1 (-0.21 to 0)	0.06	60	-0.39 (-0.52 to -0.25)	<.001	30	0.07 (-0.18 to 0.31)	0.61	11	-	-
CCI	Age adj.	103	0.04 (-0.07 to 0.14)	0.49	61	0.04 (-0.11 to 0.19)	0.60	30	0.11 (-0.09 to 0.32)	0.28	12	-0.04 (-0.18 to 0.1)	0.57
	MV adj.	97	0.04 (-0.07 to 0.15)	0.44	57	-0.04 (-0.22 to 0.14)	0.69	29	0.02 (-0.26 to 0.3)	0.91	11	-	-
GAD7	Age adj.	107	-0.04 (-0.16 to 0.09)	0.59	64	-0.16 (-0.32 to 0)	0.05	31	0.17 (-0.12 to 0.45)	0.26	12	0.01 (-0.28 to 0.3)	0.95
	MV adj.	101	-0.1 (-0.23 to 0.02)	0.10	60	-0.33 (-0.48 to -0.17)	<.001	30	0.03 (-0.27 to 0.33)	0.85	11	-	-
Z-score	Age adj.	107	-0.01 (-0.04 to 0.03)	0.65	64	-0.04 (-0.09 to 0)	0.06	31	0.05 (-0.04 to 0.13)	0.27	12	0.01 (-0.07 to 0.08)	0.88
	MV adj.	101	-0.03 (-0.06 to 0.01)	0.11	60	-0.1 (-0.14 to -0.05)	<.001	30	0 (-0.07 to 0.07)	0.90	11	-	-

Age adj.: models containing LAPS and age. MV adj.: models containing LAPS, age, body mass index (BMI), number of stressful events during past 6 months, a presence of life-threatening events (anytime in lifetime), history of physical or sexual abuse, Freiburg Mindfulness Inventory (FMI), alcohol consumption, physical activity (METs) and Berkman-Syme social index (BSSI).

* MV adjusted models did not converge.

© 2024 Żebrowska M et al. *JAMA Network Open.*

eTable 3. Association between mean Lexington attachment to pet score (LAPS) and averaged scores of depression (CESD10, K6) and anxiety (CCI, GAD7) among Mind Body Study participants who at least once reported on pet ownership and LAPS and were not married*. β_{LAPS} are effect estimates from linear regression models.

		Pet			Dog			Cat		
	**Model**	N	**β_{LAPS} (95%CI)**	**p**	**N**	**β_{LAPS} (95%CI)**	**p**	**N**	**β_{LAPS} (95%CI)**	**p value**
CES-D-10	Age adj.	33	-0.09 (-0.55 to 0.38)	0.72	14	-1.4 (-1.83 to -0.97)	<.001	15	0.78 (-0.14 to 1.7)	0.10
	MV adj.	32	-0.12 (-0.53 to 0.3)	0.58	14	-1.54 (-1.86 to -1.22)	<.001	14	0.93 (0.08 to 1.79)	0.03
K6	Age adj.	33	-0.1 (-0.38 to 0.18)	0.48	14	-0.8 (-1.16 to -0.43)	<.001	15	0.22 (-0.35 to 0.8)	0.45
	MV adj.	32	-0.13 (-0.31 to 0.06)	0.18	14	-0.94 (-1.12 to -0.76)	<.001	14	0.37 (-0.03 to 0.77)	0.07
CCI	Age adj.	33	-0.12 (-0.41 to 0.18)	0.43	14	-0.36 (-0.83 to 0.1)	0.13	15	0.15 (-0.49 to 0.79)	0.65
	MV adj.	32	-0.07 (-0.33 to 0.18)	0.58	14	-0.76 (-1.17 to -0.35)	<.001	14	-0.08 (-0.83 to 0.68)	0.84
GAD7	Age adj.	33	-0.37 (-0.81 to 0.07)	0.10	14	-1.84 (-2.35 to -1.33)	<.001	15	0.59 (-0.1 to 1.27)	0.10
	MV adj.	32	-0.41 (-0.7 to -0.13)	<0.01	14	-1.81 (-2.35 to -1.28)	<.001	14	0.79 (0.7 to 0.89)	<.001
Z-score	Age adj.	33	-0.06 (-0.17 to 0.04)	0.25	14	-0.37 (-0.51 to -0.23)	<.001	15	0.14 (-0.04 to 0.33)	0.14
	MV adj.	32	-0.06 (-0.14 to 0.01)	0.10	14	-0.44 (-0.56 to -0.33)	<.001	14	0.15 (0 to 0.31)	0.05

Age adj.: models containing LAPS and age. MV adj.: models containing LAPS, age, body mass index (BMI), number of stressful events during past 6 months, a presence of life-threatening events (anytime in lifetime), history of physical or sexual abuse, Freiburg Mindfulness Inventory (FMI), alcohol consumption, physical activity (METs) and Berkman-Syme social index (BSSI).

*Models did not converge in the mixed owners group.

© 2024 Żebrowska M et al. *JAMA Network Open.*
