# Supplementary material for: Pet Attachment and Anxiety and Depression in Middle-Aged and Older Women
Source: JAMA Netw Open. 2024 Aug 1;7(8):e2424810. doi: 10.1001/jamanetworkopen.2024.24810 (PMC11294964; doi:10.1001/jamanetworkopen.2024.24810)
Supplement: Supplement 2. — Data Sharing Statement [file jamanetwopen-e2424810-s002.pdf]

**Data Sharing Statement**

**Data**
**Data available:** Yes
**Data types:** Deidentified participant data
**How to access data:** Deidentified participant data will be made available upon reasonable request
**When available:** With publication

**Supporting Documents**
**Document types:** None

**Additional Information**
**Who can access the data:** researchers whose proposed use of the data has been approved
**Types of analyses:** For specified purpose only
**Mechanisms of data availability:** after approval of a proposal
